# Generalized crystal-storing histiocytosis with diffuse large B-cell lymphoma and monoclonal gammopathy in a Chinese elderly woman: a case report

**DOI:** 10.1186/s12885-019-5734-x

**Published:** 2019-05-29

**Authors:** Qing Tao, Wenyan Zhang, Zihang Chen, Limin Gao, Jiaqi Yan, Mi Wang, Chunxiang Xiang, Weiping Liu

**Affiliations:** 1 Department of Pathology, West China Hospital, Sichuan University, No. 37 Guoxue Alley, Chengdu, 610041 Sichuan China; 2 Department of Dermatology, West China Hospital, Sichuan University, No. 37 Guoxue Alley, Chengdu, 610041 Sichuan China

**Keywords:** Crystal-storing histiocytosis, Diffuse large B-cell lymphoma, Monoclonal gammopathy, Immunoglobulin

## Abstract

**Background:**

Crystal-storing histiocytosis (CSH) is a rare lesion characterized by sheets of crystal-laden non-neoplastic histiocytes. CSH shows a prominent association with lymphoproliferative disorders that express monoclonal immunoglobulins, mainly multiple myeloma (MM), lymphoplasmacytic lymphoma (LPL) and monoclonal gammopathy of undetermined significance (MGUS). However, no aggressive B cell lymphoma has been reported to be associated with CSH.

**Case presentation:**

A 74-year-old Chinese woman presented with multiple subcutaneous masses, abdominal pain, and fever. An IgM kappa type of monoclonal gammopathy (MG) was noted by immunofixation performed on the patient’s serum. Computed tomographic (CT) scan revealed subcutaneous masses on the left upper arm and at the waist and multiple low-density lesions in the spleen. Microscopically, sections of subcutaneous masses revealed sheets of large polygonal and spindle cells with abundant eosinophilic cytoplasm, round to ovoid eccentric nuclei, reticulate chromatin, and median nucleoli. Massive needle-shaped crystals were confined to the cytoplasm. Immunohistochemically, these crystal-containing cells were positive for CD68/PGM1, CD163, IgM, and Igκ. Meanwhile, the splenic tumour was diagnosed as diffuse large B-cell lymphoma (DLBCL), non-germinal-centre B (non-GCB) subtype (Hans algorithm). Immunohistochemistry for IgM was positive in the cytoplasm of some neoplastic cells. Immunoglobulin heavy chain (Ig*H*) gene rearrangement was detected by PCR analysis of the subcutaneous mass and the splenic tumour.

**Conclusion:**

To the best of our knowledge, this is the first case of generalized CSH with DLBCL and MG. Although the rarity of CSH and separate locations of CSH and lymphoma led to a diagnostic dilemma, the presence of MG was a clue to appreciate the relation between CSH and DLBCL. This case stressed a full investigation into the underlying lymphoproliferative disorder for integrated diagnosis and correct treatments.

## Background

Crystal-storing histiocytosis (CSH) is a rare lesion characterized by sheets of crystal-laden non-neoplastic histiocytes. Characteristic crystalline inclusions within the cytoplasm of histiocytes were first described by Glaus in 1917 [1]. The condition is still under-recognized given the rare incidence. Notably, CSH shows a prominent association with an underlying lymphoproliferative disorder, mainly MM, LPL and MGUS [2]. However, no aggressive B cell lymphoma has been reported to be associated with CSH. We report a case of CSH with DLBCL in a 74-year-old Chinese woman who presented with subcutaneous masses, abdominal pain, and fever. IgM κ paraproteinemia and proteinuria were also present. To the best of our knowledge, this is the first report of CSH with DLBCL and MG.

## Case presentation

### Medical history

**Fig. 1:**
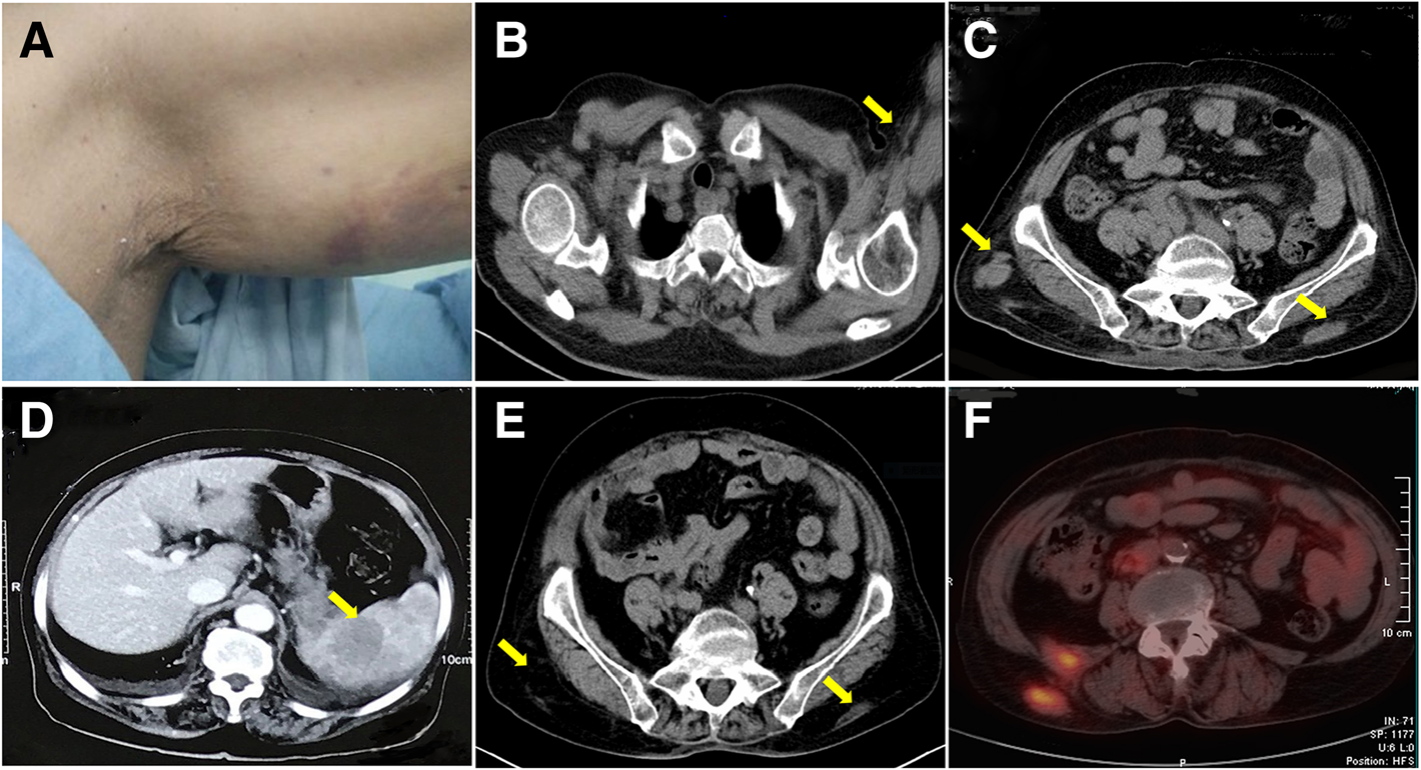
General observation and imaging examinations. (**a**): Subcutaneous mass at the left upper arm. (**b**-**d**): CT scans before the treatment. (**b**): A 2.0 cmx1.6 cm subcutaneous poor-defined tumour (arrow) at the left upper arm. (**c**): Several ill-defined soft tissue density lesions (arrows) at the waist. (**d**): Multiple low-density plaques (arrow) in the enlarged spleen by abdominopelvic contrast-enhanced CT. (**e**&**f**): Smaller lumbar masses (arrows) by PET-CT at the nine-month follow-up

**Fig. 2:**
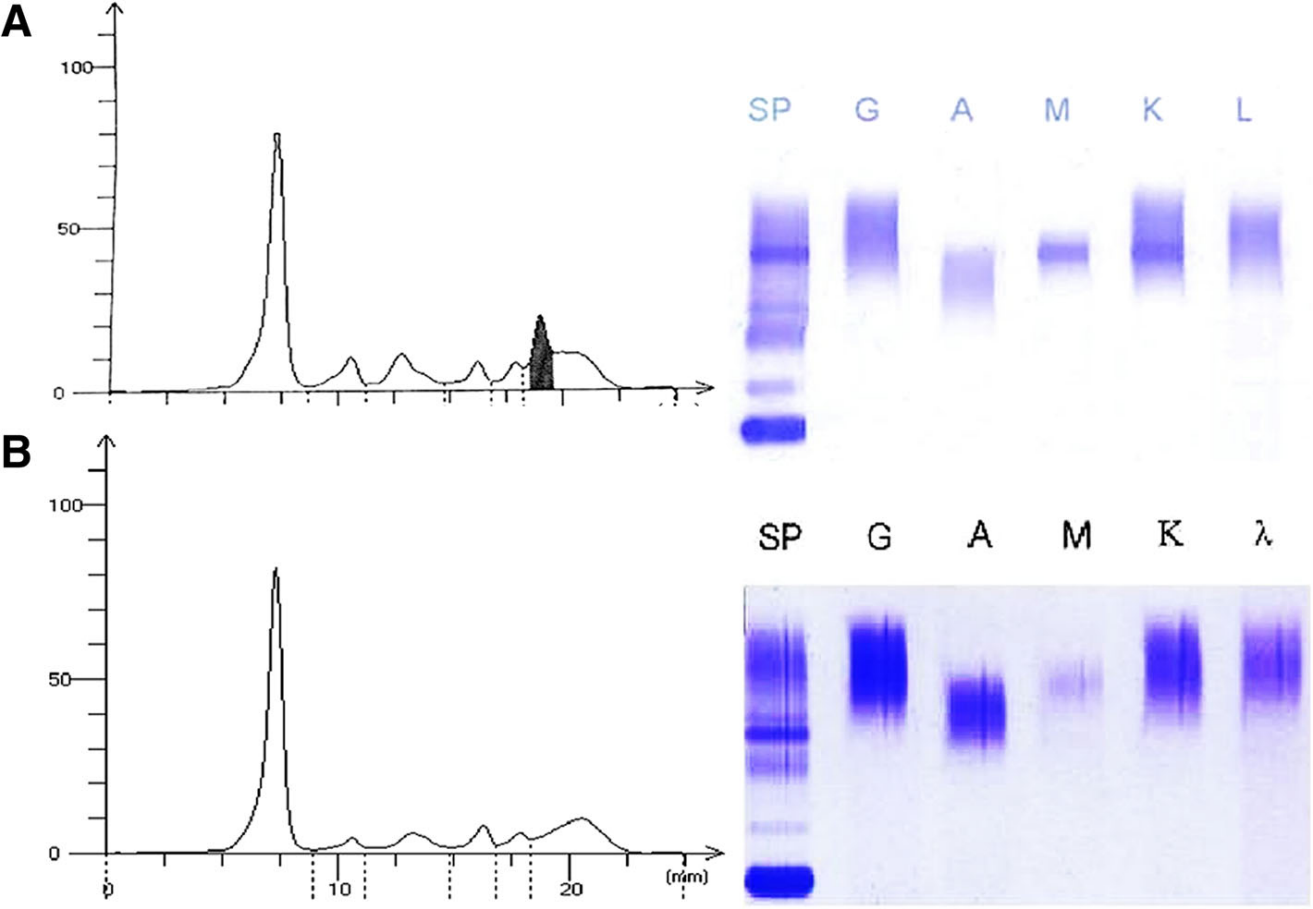
Serum protein studies. (**a**): Before the treatment, serum protein electrophoresis showed a monoclonal band, which was determined to be of the IgM κ type by immunofixation. (**b**): The paraprotein disappeared at the one-year follow-up

A 74-year-old woman was admitted with the chief complaint of subcutaneous masses, abdominal pain, and fever. Six months before the admission, the patient developed thrombocytopenia while being treated with antibiotics for pneumonia. At the time of admission, physical examination revealed a firm non-tender subcutaneous mass on the left upper arm (Fig. 1a) and two at the waist. Her abdomen was soft, but light tenderness was present in the upper abdomen without rebound tenderness. The liver and the spleen were not palpable below the costal margin. The laboratory tests results were as follows. (1) A white blood cell count of 3.48 × 10^9^/L (reference range 3.5–9.5 × 10^9^/L), an extremely low platelet count of 7.0 × 10^9^/L (reference range 100–300 × 10^9^/L) and haemoglobin of 96 g/L (reference range 115–150 g/L) were noted. (2) Serum protein electrophoresis showed a monoclonal band, which was determined to be of the IgM κ type by immunofixation (Fig. 2a). Serum protein quantitative analysis revealed 10.30 g/L IgM, 20.10 g/L kappa light chain, 7.04 g/L lambda light chain and kappa/lambda = 2.86 (reference range 2.20,13.00 and 6.50 g/L and reference ratio of 2.56, respectively). All the other immunoglobin (Ig) levels were normal. (3) Urinalysis result was 1 + (0.5 g/L) (reference range negative, 0 g/L) for protein. (4) Bone marrow aspiration and flow cytometry (FCM) analysis were normocellular. Computed tomographic (CT) scan showed a 2.0 cmx1.6 cm subcutaneous mass on the left upper arm and several ill-defined soft tissue lesions at the waist along with intra-abdominal lymphadenopathy and moderate splenomegaly (Fig. 1b&c). Abdominopelvic contrast-enhanced CT displayed multiple low-density plaques in the spleen (Fig. 1d). The patient underwent a needle biopsy of the subcutaneous mass on the left arm in a local hospital. After her admission, the subcutaneous mass at the right waist was excised, and laparoscopic splenectomy was performed.

### Pathologic findings

**Fig. 3:**
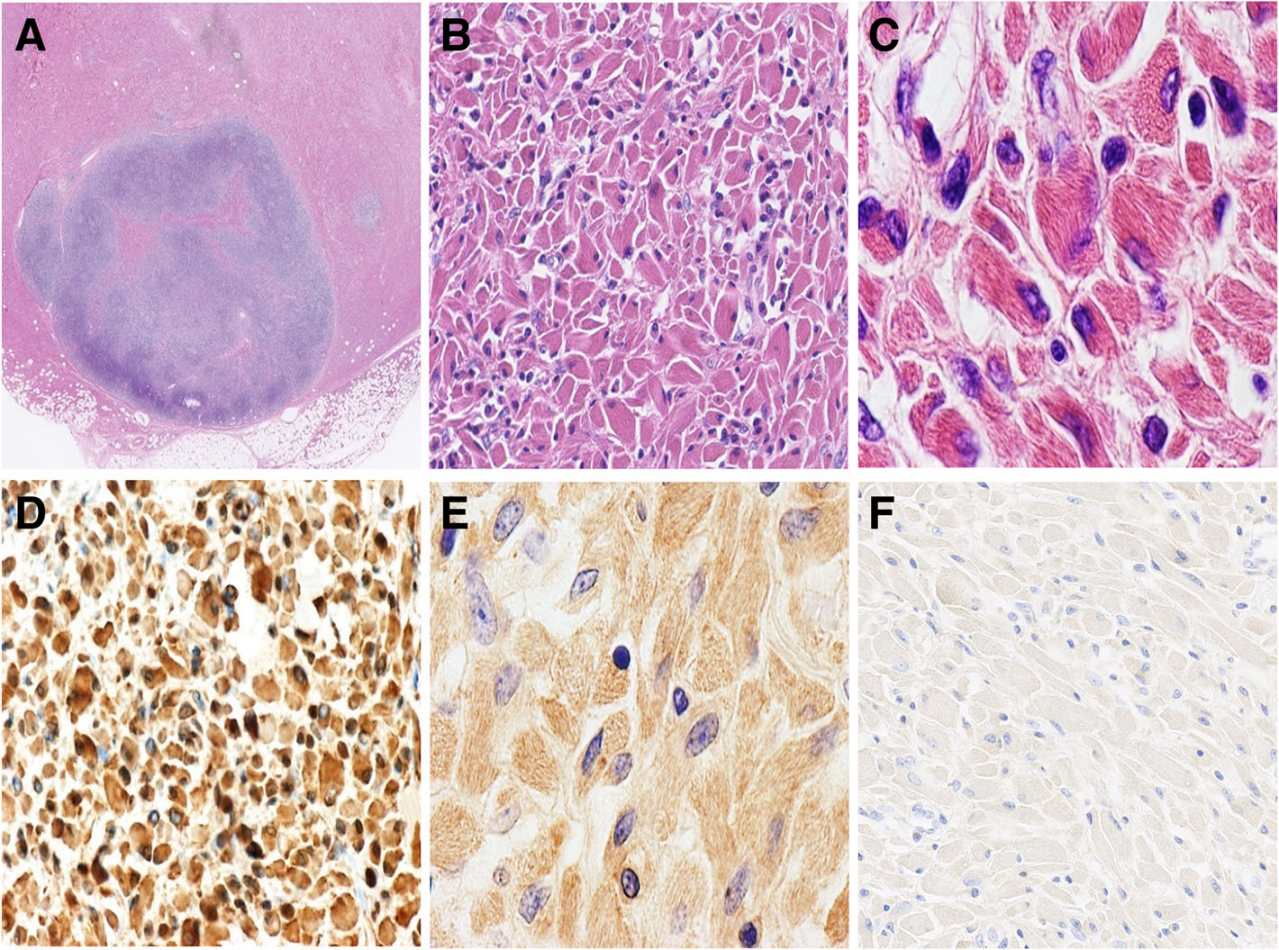
Microscopic appearance and immunophenotypes of the subcutaneous masses at the waist. (**a**): The lesion is restricted in the subcutaneous tissue and an intact lymph node infiltrated by crystal-laden histiocytes (the pink area), HE × 10. (**b**): Sheets of large polygonal and spindle histiocytes, HE × 400. (**c**): Needle-shaped crystals within the cells, HE × 1000. (**d**): CD68 × 400; (**e**): IgM × 1000; (**f**): IgG × 400

**Fig. 4:**
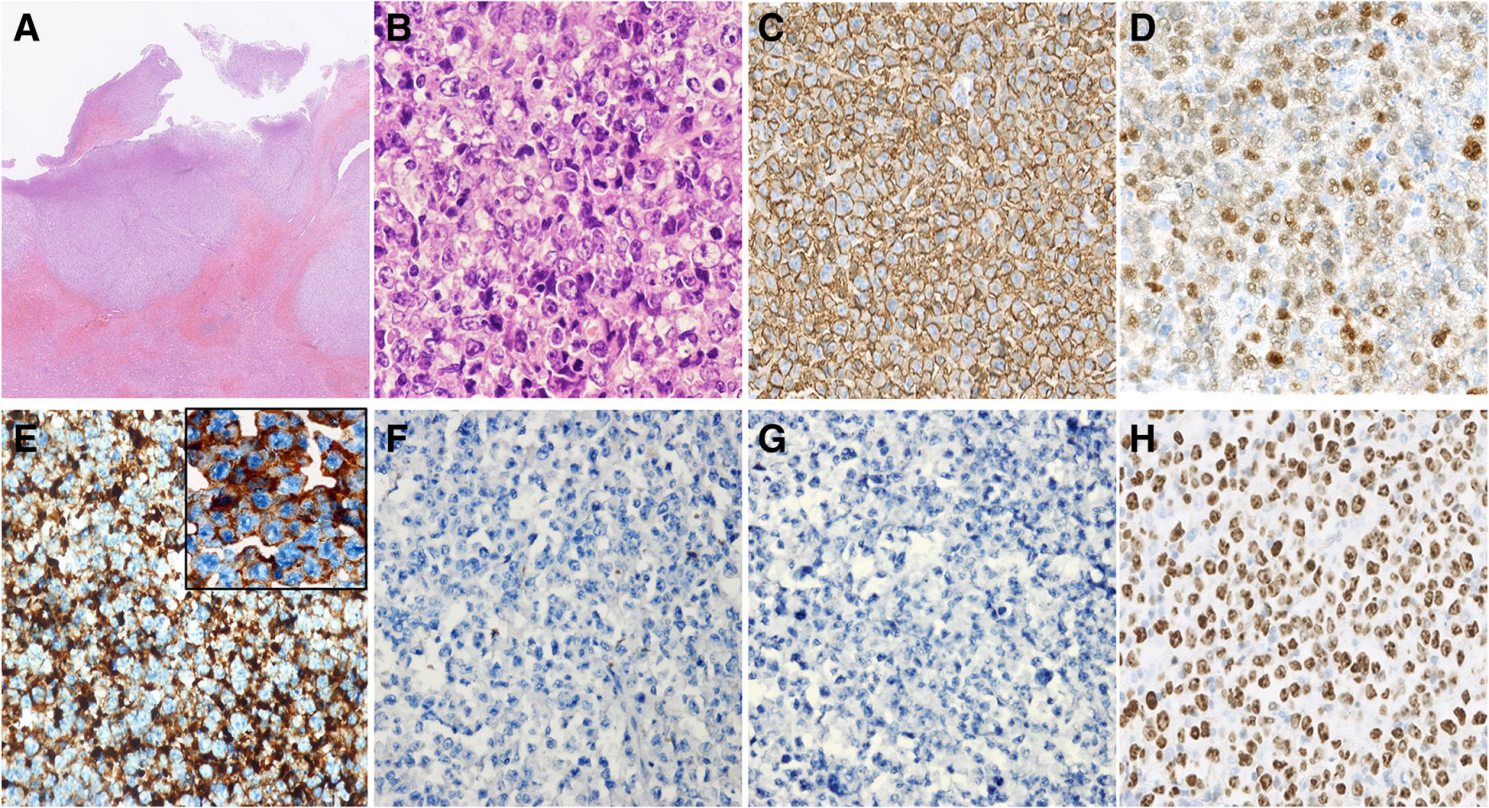
Microscopic appearance and immunophenotypes of the tumour at the splenic hilum. (a-h): Formalin-fixed paraffin-embedded splenic tumour sections (**a**): A focal infiltration of lymphocytes, HE × 10. (**b**): Diffuse large centroblast-like cells with a round, oval or irregular nuclei, distinct nucleoli, and scant cytoplasm. Frequent mitoses were seen. HE × 400. (**c-h**) original magnification, × 400. (**c**): CD20; (**d**): MUM-1; (**e**): IgM (Insert × 1000); (**f**): IgG; (**g**): CD138; (**h**): Ki-67

(1) Macroscopically, the resected lumbodorsal specimen was a firm grey-brown mass measuring 5.5 cm × 3.0 cm × 1.5 cm in volume. Microscopic examination revealed diffuse proliferation in the subcutaneous tissue, which was composed of large polygonal and spindle histiocytes with abundant eosinophilic cytoplasm, round to ovoid eccentric nuclei, reticulate chromatin and median nucleoli (Figs. 3a&b). Needle-shaped crystals were confined to the cytoplasm, some of which were in parallel arrays (Fig. 3c). These crystal-containing cells were located in and around an intact lymph node measuring 0.8 cm in maximal dimension. The paracortical area of the lymph node was expanded by extensive infiltration of the crystal-containing cells and mature plasma cells (Fig. 3a). These crystal-containing cells were strongly positive for CD68/PGM1 (Fig. 3d) and CD163 and negative for S-100, desmin, SMA, MSA, and myogenin. Immunostain for IgM heavy chain was strong and diffuse (Fig. 3e). Immunostain for the κ light chain was relatively weak. Immunostains for the IgG heavy chain and λ light chain were negative (Fig. 3f). The plasma cells were characterized by CD38+, MUM1+, and kappa > lambda. Congo red staining was negative. BIOMED-2 multiplex PCR analysis showed diversity-joining gene segments of immunoglobin heavy chain (Ig*H*-DH-JH) gene rearrangement (the monoclonal peak was at approximately 400 bp) and Ig*κ* gene rearrangement. The *MYD88* L265p mutation was not detected. The diagnosis of “CSH associated with Ig*H* and Ig*κ* rearrangements” was made with a comment that an underlying lymphoproliferative disorder should be suspected.

(2) Review of the needle biopsy of the mass on the left upper arm identified a similar cell population as the one at the waist, which was characterized by sheets of large rhabdoid cells with abundant deeply eosinophilic cytoplasm containing massive crystalline materials, eccentric irregular nuclei, and inconspicuous nucleoli.

(3) The spleen was enlarged to 15 cm × 10 cm × 8 cm and weighed 198 g with a smooth surface. Serial sectioning showed a grey irregular solid tumour measuring 3 cm × 1.5 cm × 1.3 cm at the splenic hilum mixed with adipose tissue and focal necrotic areas. Microscopic examination revealed diffuse infiltration of large cells with a round, oval or irregular nuclei, distinct nucleoli and scant cytoplasm, centroblast like (Fig. 4a-b). Frequent mitoses were observed (Fig. 4b). Immunohistochemically, the neoplastic cells were CD20+, Bcl6+, MUM1+, CD3p-, CD10-, CyclinD1- and CD43- (Fig. 4c-d). IgM was strongly positive in the cytoplasm of some neoplastic cells, while IgG, CD38, and CD138 were negative (Fig. 4e-g). The Ki-67 index was 80% (Fig. 4h). Fluorescence in situ hybridization (FISH) suggested *BCL-6* rearrangements but the absence of dysregulations of *BCL2* and *MYC*. In situ hybridization for Epstein-Barr virus-encoded RNA (EBER-ISH) was negative. A monoclonal peak of the Ig*H* gene was detected at approximately 420 bp by commercial BIOMED-2 multiplex PCR system. These findings conformed to a diagnosis of DLBCL, non-GCB subtype (Hans algorithm). The pancreas was involved, while the liver was not. CSH lesions were not found in the spleen and the regional lymph nodes.

### Treatment and follow-up

After splenectomy, the platelet count increased to 128 × 10^9^/L. The patient was administered eight courses of rituximab, cyclophosphamide, doxorubicin, vincristine, and prednisone (R-CHOP) and was in partial remission (PR) 9 months after diagnosis. Positron emission tomography-computed tomography (PET-CT) scan showed resolution of the mass at the left arm, smaller lumbar subcutaneous masses and intraperitoneal lymphadenopathy (Fig. 1e-f). However, serum paraprotein levels remained elevated (IgM 4.01 g/L). Thus, the patient received four cycles of rituximab (600 mg/m^2^ day 1) plus lenalidomide (25 mg/m^2^ days 1–14 q28), and the MG was not visible (Fig. 2b) at a one-year follow-up.

## Discussion and conclusions

CSH is a rare disorder diagnosed only on the basis of pathologic features, but its prominent association with lymphoproliferative disorders has been expounded in the literature. To date, 140 cases of CSH have been reported according to a PubMed search of the English literature. Patients can be classified into localized CSH (L-CSH) and generalized CSH (G-CSH) based on the number of the foci. G-CSH, which entails the involvement of two or more organs/systems, may present with a rapid clinical course and portend a worse prognosis [2]. Recently, Fang H et al. reported 8 cases of CSH with bone marrow involvement and reviewed the English literature (total of 131 cases) from 1987 to 2017 [3]. Of the 131 cases of CSH, 100 cases (76%) were associated with a lymphoproliferative disorder, mainly MM, LPL, and MGUS. Thirty-five cases (27%) were accompanied with B-cell lymphoma, but none of these lymphomas were aggressive B-cell lymphoma. To the best of our knowledge, this is the first case of CSH with DLBCL.

DLBCL was mentioned in 5 patients with CSH in the literature (Table 1), and four of these cases transformed from or were accompanied with low-grade B-cell lymphoma [4–7]. Although a concurrent low-grade B-cell component cannot be completely excluded in the unbiopsied sites of abdominal lymphadenopathy in the present case, biopsies of multiple anatomic sites failed to obtain any evidence of low-grade B-cell lymphoma, and normal bone marrow biopsy and the undetected *MYD88* mutation contributed to the exclusion of LPL. In addition, there was no history of lymphoproliferative disorders in our patient. Kawano et al. reported a case of pulmonary L-CSH without lymphoproliferative disorders in a patient with a history of gastric DLBCL [8]. In that report, the gastric DLBCL retained CR without evidence of recurrence, and the PCR analysis with stomach and lung tissues did not reveal the same clone. Thus, the ultimate diagnosis was L-CSH without lymphoproliferative disorders. Similarly, in the present case, the CSH lesions and the DLBCL involved different anatomic sites respectively and exhibited unrelated Ig*H* gene monoclonal peaks in PCR analysis. However, the presence of MG and the generalized CSH raised concerns for a systematic association. As expected, subsequent immunohistochemistry showed strong IgM staining in some DLBCL cells without plasmablastic differentiation. These neoplasm cells may be responsible for the IgM type of MG and the generalized CSH. In addition, the level of serum paraprotein changed with the response to chemotherapy, and this finding could point to the unusual origin of immunoglobulins. Taking these findings together, we preferred a diagnosis of CSH with DLBCL rather than a CSH independent of the DLBCL and MG.

**Table 1:** Summary of CSH cases coexistent with DLBCL

Report/Year	Age/sex	Symptoms / History	Diagnosis	Ig type	Sites of CSH	Clinical course
Kaufmann/1996 [5]	61/F	Subcutaneous masses	G-CSH with LPL in transformation to a large cell lymphoma	IgM kappa IgG lambda	Skin, LN	Not reported
Jones/1999 [4]	70/M	Splenomegaly	G-CSH with LPL	IgM kappa	BM, LN	Transformed to DLBCL at 24mos
Thorson/2000 [7]	77/F	Bilateral cervical lymphadenopathy	G-CSH with Low-grade monocytoid B-cell lymphoma	lambda	Parotid and submandibular glands, LN	Transformed to DLBCL at 8ys
Li JJ /2015 [6]	80/F	Bilateral periorbital swelling	L-CSH with WM	IgM kappa	Skin	Transformed to DLBCL at 6ys and died of disease
Kawano/2013 [8]	80/M	Asymptomatic/ Gastric DLBCL 13 years ago(CR)	L-CSH without LP-PCD	None	Lung	Live free of disease at a 23mos follow-up
Present case	74/F	Abdominal pain, fever, subcutaneous masses	G-CSH with DLBCL	IgM kappa	Soft tissue, LN	Live with disease at a 1y follow-up

F: Female; M: Male; L-CSH: Localized crystal-storing histiocytosis; G-CSH: Generalized crystal-storing histiocytosis; DLBCL: Diffuse large B-cell lymphoma; LPL: Lymphoplasmacytic lymphoma; WM: Waldenström’s macroglobulinemia; LP-PCD: Lymphoproliferative or plasma cell disorder; CR: Complete remission; LN: Lymph node; BM: Bone marrow; mo: Month; y: Year

The mechanism of crystal formation is unclear. It is hypothesized that a combination of overproduction and conformational alterations in the immunoglobin light chains leads to crystallization, impaired enzymatic degradation by histiocytes, and crystal accumulation within histiocytes [9–11]. Afterwards, Kanagal-Shamanna et al. observed that the sole representation of the variable region of immunoglobin heavy chains contributed to abnormal immunoglobin structure by proteomic methods, expanding the proposed hypothesis [12]. The present case posed a diagnostic challenge due to the separate locations of the CSH and DLBCL, and gene-rearrangement studies showed that the variable region encoded by Ig*H*-DH-JH gene segments may be related to the crystallization. More molecular analyses are required to clarify the mechanism.

It is morphologically difficult to distinct CSH from other histiocytosis and sedimentary lesions, such as xanthogranuloma, Langerhans cell histiocytosis, granular cell tumour, fibrous histiocytoma, amyloidosis, and Gaucher’s disease. On low-power images, CSH is characterized by sheets of polygonal or spindle-shaped histiocytes with abundant eosinophilic cytoplasm. On high-power images, refractive needle-like crystalline material filled the cytoplasm. Immunohistochemical analysis is useful to the differential diagnosis. In our case, CD68 and CD163 immunohistochemical stains confirmed that the large, pink cells were histiocytes. S100 protein immunohistochemical stain excluded the possibility of granular cell tumour and Langerhans cell histiocytosis. Congo red excluded amyloidosis, and desmin, MSA and myogenin were performed to exclude adult rhabdomyoma. There were a few cases of CSH mimicking adult rhabdomyoma given the similar morphologic features and location in soft tissue [13–15]. In patients with a malignant tumour, CSH may be clinically diagnosed as metastatic carcinoma [16].

Finally, the treatment and prognosis of patients with CSH vary depending on the associated disease [2]. Regarding DLBCL, MG was reported as a poor prognostic marker in patients with the non-GCB type [17]. The outcome after R-CHOP was unsatisfactory in IgM-secreting DLBCL patients, while immunochemotherapy combined with bortezomib or lenalidomide seemed to be more effective [18]. In our patient, the general status improved with R-CHOP. However, the serum paraprotein remained elevated, and multiple lymph node involvement remained detectable. Lenalidomide accelerated the disappearance of paraprotein and might play a positive role in this subset of patients. Further accumulation of cases is essential to expound the treatment and prognosis of CSH and DLBCL with MG.

In conclusion, the presented case is the first to describe G-CSH concomitant with DLBCL and MG. The rarity of CSH and separate locations of CSH and DLBCL contributed to the complexity of the diagnostic approach. This case highlights an unusual origin of immunoglobulins and the overproduction of the immunoglobulins may promote the G-CSH. Immunohistochemical analysis can assist in differential diagnosis and classification. Once a pathologic diagnosis of CSH is confirmed, subsequent investigations for the underlying lymphoproliferative disorders are required, such as a detailed clinical history, serum and urine protein studies, imaging examinations and bone marrow biopsy.

## Data Availability

All the data supporting the findings are presented within the manuscript and supplementary data.

## Abbreviations

BM: Bone marrow
CR: Complete remission
CSH: Crystal-storing histiocytosis
CT: Computed tomographic
DH-JH: diversity-joining gene segments of the immunoglobin heavy chain
DLBCL: Diffuse large B-cell lymphoma
EBER-ISH: In situ hybridization for Epstein-Barr virus-encoded RNA
F: Female
FCM: Flow cytometry
FISH: Fluorescence in situ hybridization
G-CSH: Generalized crystal-storing histiocytosis
Ig: Immunoglobulin
Ig*H*: Immunoglobulin heavy chain
L-CSH: Localized crystal-storing histiocytosis
LN: Lymph node
LPL: Lymphoplasmacytic lymphoma
LP-PCD: Lymphoproliferative or plasma cell disorder
M: Male
MG: Monoclonal gammopathy
MGUS: Monoclonal gammopathy of undetermined significance
MM: Multiple myeloma
mo: Month; y:Year
non-GCB: non-germinal-center B
PCR: Polymerase chain reaction
PET-CT: Positron emission tomography–computed tomography
PR: Partial remission
WM: Waldenström’s macroglobulinemia
